# Supplementary material for: Farm living and risk of asthma, atopic eczema, respiratory and food allergy: protocol for a systematic review and meta-analysis
Source: BMJ Open. 2021 Dec 30;11(12):e048736. doi: 10.1136/bmjopen-2021-048736 (PMC8719174; doi:10.1136/bmjopen-2021-048736)
Supplement: Supplementary data [file bmjopen-2021-048736supp001.pdf]

APPENDIX 1

PUBMED SEARCH STRATEGY

CONCEPT 1: Farm (living)

"Farms"[Mesh] OR Farmland OR "farm living" OR farm* OR "crop farmer" OR "crop farmers" OR "crop farm" OR "crop farming" OR husbandar* OR animal* OR "animal rearing" OR farmstead* OR "ranch" OR "plantation" OR "croft" OR "shamba".

CONCEPT 2: Asthma and allergy outcomes

Asthma[mh] OR Asthma, Exercise-Induced[mh] OR Respiratory Hypersensitivity[mh] OR bronchial asthma*[tiab] OR exercise-induced asthma*[tiab] OR asthma*[tiab] OR respiratory hypersensitivit*[tiab] OR airway hyper responsiveness[tiab] OR airway hyper-responsiveness[tiab] OR respiratory hyper responsiveness[tiab] OR respiratory hyper-responsiveness[tiab] OR wheez*[tiab] OR Hypersensitivity[mh] OR Hypersensitivity, Immediate[mh] OR Hypersensitivity, Delayed[mh] OR Allergy and Immunology[mh] OR immediate hypersensitivit*[tiab] OR delayed hypersensitivit*[tiab] OR hypersensitivit*[tiab] OR IgE-mediated hypersensitivit*[tiab] OR type I hypersensitivit*[tiab] OR type IV hypersensitivit*[tiab] OR atopic sensitization[tiab] OR atop*[tiab] OR allergic sensitization[tiab] OR allergic disease*[tiab] OR allerg*[tiab] OR Dermatitis, Atopic[mh] OR Eczema[mh] OR Angioedema[mh] OR Anaphylaxis[mh] OR Urticaria[mh] OR atopic dermatitis[tiab] OR dermatitis[tiab] OR atopic eczema[tiab] OR eczema[tiab] OR urticari*[tiab] OR anaphyla*[tiab] OR quincke edema[tiab angioedema[tiab] OR hives[tiab] OR Food Hypersensitivity[mh] OR food hypersensitivit*[tiab] OR food allerg*[tiab] OR egg allerg*[tiab] OR egg hypersensitivit*[tiab] OR milk allerg*[tiab] OR milk hypersensitivit*[tiab] OR shellfish allerg*[tiab] OR shellfish hypersensitivit*[tiab] OR wheat allerg*[tiab] OR wheat hypersensitivit*[tiab] OR nut allerg*[tiab] OR nut hypersensitivit*[tiab] OR peanut allerg*[tiab] OR peanut hypersensitivit*[tiab] OR Rhinitis, Allergic[mh] OR Rhinitis, Allergic, Seasonal[mh] OR Rhinitis, Allergic, Perennial[mh] OR Rhinitis[mh] OR Conjunctivitis, Allergic[mh] OR allergic rhinoconjunctiviti*[tiab] OR rhinoconjunctiviti*[tiab] OR allergic rhiniti*[tiab] OR rhiniti*[tiab] OR seasonal allergic rhiniti*[tiab] OR perennial allergic rhiniti*[tiab] OR allergic conjunctiviti*[tiab] OR hay fever[tiab] OR hayfever
