# Supplementary material for: Farm living and risk of asthma, atopic eczema, respiratory and food allergy: protocol for a systematic review and meta-analysis
Source: BMJ Open. 2021 Dec 30;11(12):e048736. doi: 10.1136/bmjopen-2021-048736 (PMC8719174; doi:10.1136/bmjopen-2021-048736)
Supplement: Author's manuscript [file bmjopen-2021-048736.draft_revisions.pdf]

BMJ Open

BMJ Open is committed to open peer review. As part of this commitment we make the peer review history of every article we publish publicly available.

When an article is published we post the peer reviewers' comments and the authors' responses online. We also post the versions of the paper that were used during peer review. These are the versions that the peer review comments apply to.

The versions of the paper that follow are the versions that were submitted during the peer review process. They are not the versions of record or the final published versions. They should not be cited or distributed as the published version of this manuscript.

BMJ Open is an open access journal and the full, final, typeset and author-corrected version of record of the manuscript is available on our site with no access controls, subscription charges or pay-per-view fees (http://bmjopen.bmj.com).

If you have any questions on BMJ Open's open peer review process please email info.bmjopen@bmj.com

BMJ Open

Farm living and risk of asthma and allergy; protocol for a systematic review and meta-analysis.

Journal:	*BMJ Open*
Manuscript ID	bmjopen-2021-048736
Article Type:	Protocol
Date Submitted by the Author:	10-Jan-2021

Complete List of Authors:	Kuya, Johnstone; Africa Development and Strategy Centre, Public health<br>Omoronyia, Ogban; University of Calabar, Community Medicine<br>Fokukora, Theopista; Cavendish University, Department of Public Health<br>Emmanuel, Aduroja Posi; University of Ibadan, Department of Health Promotion and Education<br>Ewemooje, Olusegun; Federal University of Technology Akure, Department of Statistics<br>Soyege, Adewumni ; Babcock University, Department of Public Health<br>Ngo, Valery; University of Gothenburg, Department of Global Health<br>Anyiam, Felix Emeka; University of Port Harcourt<br>Ackah, Josephine Akua; University of Cape Coast, Department of Population and Health<br>Ossai-Chidi, Linus; University of Port Harcourt Teaching Hospital, Department of Medical Microbiology<br>Manuel, Beatriz; Eduardo Mondlane University, Department of Community Health<br>Emmanuel, Okondu Ogechukwu; Babcock University, Department of Public Health<br>Nwaru, Bright; University of Gothenburg

Keywords:	Asthma < THORACIC MEDICINE, Allergy < THORACIC MEDICINE, RESPIRATORY MEDICINE (see Thoracic Medicine), PUBLIC HEALTH, EPIDEMIOLOGY

SCHOLARONE™
Manuscripts

Farm living and risk of asthma and allergy; protocol for a systematic review and meta-analysis.

Johnstone Kuya[1], Ogban Omoronyia[2], Theopista Fokukora[3] Aduroja Posi Emmanuel [4] Olusegun Sunday Ewemooje[5] Adewumni O. Soyege [6], Valery Ngo[7], Felix Emeka Anyiam [8], Josephine Akua Ackah[9], Linus Ossai-Chidi[10], Beatriz Manuel[11], Okondu Ogechukwu Emmanuel [12], Bright I. Nwaru[13]

Author affiliations
[1]Africa Development and Strategy Centre, Nairobi, Kenya.
2 Department of Community Medicine, University of Calabar, Calabar, Nigeria
[3]Department of Public Health, Faculty of Science and Technology, Cavendish University, Kampala, Uganda
[4]Department of Health Promotion and Education, University of Ibadan, Ibadan, Nigeria.
[5]Department of Statistics, Federal University of Technology, Akure, Nigeria.
[6]Department of Public Health, School of Public and Allied Health, Babcock Univeristy, Ilishan-Remo, Nigeria.
[7]Department of Global Health, School of Public Health and Community Medicine, The Sahlgrenska Academy, University of Gothenburg, Gothenburg, Sweden.
[8]Centre for Health and Development (CHD), University of Port Harcourt, Port Harcourt, Nigeria
[9] Department of Population and Health, Faculty of Social Sciences, University of Cape Coast, Ghana
[10] Department of Medical Microbiology, University of Port Harcourt Teaching Hospital, Rivers State. Nigeria
[11] Department of Community Health, Faculty of Medicine, Eduardo Mondlane University, Maputo Mozambique.
[12] Department of Public Health, School of Public and Allied Health, Babcock Univeristy, Ilishan-Remo, Nigeria
[13]Krefting Research Centre, Institute of Medicine, Sahlgrenska Academy, University of Gothenburg, Gothenburg, Sweden

Correspondence:

Bright Nwaru
Krefting Research Centre
Institute of Medicine
University of Gothenburg
+46 31 786 6718
bright.nwaru@gu.se

For peer review only - http://bmjopen.bmj.com/site/about/guidelines.xhtml

ABSTRACT

Introduction: Asthma and allergic disorders are of global concern and risk for their development results from the interplay of genetic and environmental factors. Among numerous environmental factors identified to influence the disease risk, the role of exposure to a farming environment has gain interest in recent times, but the underlying evidence is conflicting. We aim to comprehensively identify, critically appraise, and synthesize evidence from studies investigating the association between farm living and risk of asthma and allergic diseases in children and adults.

Methods and Analysis: We will identify relevant analytical observational epidemiological studies, including cross-sectional, case-control and cohort studies, by searching Pubmed, Cochrane Library, Google Scholar, CINAHL, WHO Global Health Library, Web of Science, Scopus, and EMBASE. Screening of identified records, data extraction from eligible studies and risk of bias assessment of eligible studies will be independently undertaken by two reviewers, with arbitration by a third reviewer. The Effective Public Health Practice Project will be employed for the risk of bias assessment. Estimates from studies judged to be clinically, methodologically and statistically homogeneous will be synthesised using random-effects meta-analysis. Heterogeneity will be assessed using the I-squared statistic.

Primary outcomes measures: We will consider objectively-measured or self-reported asthma, atopic dermatitis/eczema, allergic rhinitis, wheeze, and food allergy.

Ethics and dissemination: As this study is based solely on the published literature, no ethics approval is required. The study findings will be presented at scientific meetings related to the field of asthma and allergy and will be published in an international peer-reviewed scientific journal.

PROSPERO registration number: CRD42020208805

Strengths and Limitation of this Study:

- Findings from this systematic review will provide a comprehensive and most contemporaneous synthesis of the underlying evidence linking exposure to a farming environment to risk of asthma and allergic diseases since the latest publication of the previous reviews was five years ago.
- This systematic review protocol follows the Preferred Reporting Items for Systematic Review and Meta-Analysis Protocols guidelines.
- The identification of studies from leading medical and public health databases, with no geographical or language limitations, will advance the import of this evidence synthesis across settings.
- We anticipate that the definition of farm exposure and asthma and allergic outcomes will vary between studies, thus posing a challenge in deriving pooled estimates of effect.

KEYWORDS: Systematic review; meta-analysis, Asthma, wheeze, atopic dermatitis, atopic sensitization, farm living, farming, allergic rhinitis, food allergy.

For peer review only - http://bmjopen.bmj.com/site/about/guidelines.xhtml

BACKGROUND

Globally, there is a high morbidity due to asthma and allergic diseases[1]. This group of immunologic conditions have continued to contribute significantly to the burden of disease and years lost due to disability in diverse global settings[1]. Currently, an estimated 339 million people have asthma, which represents the most common chronic disease among children[2]. The development of asthma and allergic diseases results from the interplay of genetic and environmental factors[3].

Numerous environmental factors linked to the risk of asthma and allergic diseases have been identified, among which include household exposures (e.g., dust mites, household dampness, indoor air pollution, moulds, and domestic pets) and non-household exposures (e.g., respiratory infections, tobacco smoke, outdoor air pollution, and exposure to a farming environment)[4]. However, findings from available studies on the influence of these environmental factors are conflicting[5-7]. With specific focus on the role of the farming environment, over the last three decades, several studies have reported protection for atopy, allergic disease, and asthma[8], while some studies have also reported that exposure to the farming environment may increase the risk of the onset of the diseases[9,10]. While a majority of these studies have focused on exposure to farming environment during early childhood and asthma and allergy in childhood, there are also some studies in adults.

The amount and variety of microbial exposure from farm living is thought to be a key mechanism through which the farming environment influences the risk of asthma and allergies[11]. Greater microbial exposure, characteristic of the more unhygienic farm environment, stimulates the development of a more effective immune system[12]. Microbial exposure helps the immune system to learn to efficiently avoid over-reaction to potentially harmless substances that may have been perceived as allergens[11]. However, the effect of microbial exposure may differ according to farm living environment or farming activities[13]. A large-scale multicentre cross-sectional study among rural school children in five European countries found that pig farming, haying, consumption of farm milk, and dwelling in the farm were associated with substantially reduced risk of asthma compared to wheeze[14]. The high proportion of whey protein found in farm milk, is thought to initiate the process of more effective regulation of the immune system to prevent over-reactivity to diverse substances[15]. Furthermore, sialic acid, more commonly found in farm animals and their products, is thought to contribute to antibody-mediated regulation of inflammatory reactions to substances[16]. A number of systematic reviews have been published that summarized the available evidence linking farming environment to risk of asthma and allergy. Dicks and colleagues[17, 18] in their review

For peer review only

revealed that habitation of children in an area with intensive farming was associated with an increased risk of being asthmatic. The review considered asthma and wheeze as the only outcomes, but importantly, it was based searches made only in Pubmed and was undertaken by only one person. Without consideration of other allergic outcomes other than asthma and wheeze does not give a comprehensive overview of the underlying evidence. Searching only one database, of several databases that collect scientific research also indicates that the generated evidence is limited, given that the overlap between Pubmed and other research databases is only partial. A systematic review should provide the highest evidence on a research topic and therefore the process needs to be transparent and reproducible, with at least two reviewers going through the processes of the review being a mandatory requirement; however, Genuneit[19] failed in this aspect as it was undertaken only by one person. The systematic review by Campbell and colleagues[20] focused only on objective measures of atopy (based on skin prick test [SPT] and immunoglobulin E [IgE] measurements) and was also based on studies retrieved only from Pubmed. Similar limitations affected this review, in that it investigated a small sub-set of atopic outcomes and performed limited search of available literature.

To gain a clearer and comprehensive appreciation of the underlying evidence linking exposure to a farming environment to risk of asthma and allergic diseases, the noted limitations in previous systematic reviews need to be overcome: there should be an exhaustive set of outcomes included; there should be an exhaustive search of the literature; and the review processes should be transparent and reproducible, with at least two people doing review. Moreover, since the publication of the previous reviews, the latest published five years ago, there have been several studies now published on the topic. These studies need to be integrated into the evidence synthesis in order to provide a contemporaneous synthesis of the evidence base.

AIMS AND OBJECTIVES

By overcoming limitations of previous systematic reviews, we aim to comprehensively identify, critically appraise, and synthesize evidence from studies investigating the association between farm living and risk of asthma and allergic diseases in children and adults.

Specific objectives are to synthesize the evidence on:

1. The association between farm living and the risk of onset of asthma and allergic diseases in children and adults.

For peer review only - http://bmjopen.bmj.com/site/about/guidelines.xhtml

2. The association between farm living and the risk of outcomes (e.g. indicators of exacerbation and severity) of asthma and allergic diseases in children and adults with already established disease.
3. Whether the association between farm living and risk of asthma and allergy depends on the type of farming environment (eg pig farmers vs cattle rearers, small vs large-scale farmers).
4. Whether the association between farm living and risk of asthma and allergy differs between geographical regions of the world (e.g. high income vs low and middle income countries).

METHODS

Study eligibility criteria

Type of studies: We will include analytical observational epidemiological studies (cross-sectional, case-control and cohort studies). We will exclude clinical case studies, case-series discussion papers, non-research letters and editorials, and animal studies.

Types of participants

We will include studies that included participants of any age, in which the association between farm living and asthma and allergic diseases have been investigated, regardless of gender, age and nationality.

Types of exposure

Studies that have investigated the role of farm living, regardless of the type of farm environment and the place of farming (rural versus urban). We will not consider the specific constituents of the farm, e.g. bio aerosols.

Types of outcome measures

As primary outcomes, we will consider objectively-measured or self-reported asthma, atopic dermatitis/eczema, allergic rhinitis, wheeze, and food allergy. Our secondary outcomes will include atopic sensitization measured by skin prick test or antigen-specific immunoglobulin E; indicators of disease severity and measures of quality of life; asthma exacerbations; use of medications for respective allergic diseases and asthma; hospitalisation for asthma; indicators of airway function including peak expiratory flow, forced expiratory volume in 1 second, forced vital capacity, forced expiratory flow rate, or other pulmonary function tests [oscillometry or exhaled nitric oxide analysis]).

For peer review only

Information sources

Database searches

Relevant studies/articles from onset of the following electronic databases till date will be sought from: Pubmed, Cochrane Library, Google Scholar, CINAHL, WHO Global Health Library, Web of Science, Scopus, and EMBASE. There will be no restriction on language and we will endeavor to tranalate papers not published in English.

Search strategy

Search strategies covering concepts and synonyms of farm living and the study outcomes (refer to the appendix of search strategies) will be adapted to search the other databases.

Screening of retrieved literature

The literature retrieved from the databases will be transferred to Endnote for removal of duplicate papers; thereafter the papers will be exported to Rayan for further screening. Two reviewers will separately evaluate the titles and abstracts of the de-duplicated articles based on the study inclusion and exclusion criteria. The full texts of eligible articles will be collected and further critically reviewed by the two reviewers for inclusion. Any differences between the two reviewers in the evaluation processes will be settled by consensus, and a third reviewer will arbitrate any disagreement. The Preferred Reporting Items for Systematic Reviews and Meta-Analysis PRISMA flow chart will be used to report the screening process.

Registration and reporting

This study protocol has been registered with the University of York Centre for Reviews and Dissemination International prospective register of systematic reviews (PROSPERO number CRD42020208805). The protocol is reported according to the PRISMA-P guidelines for reporting of systematic review protocols, awaiting registration number.

Data extraction

A standardized data extraction form will be developed and will be used by at least two members of the review team to independently extract relevant study data from the full-text papers included in the systematic review. Before full use with all included studies, the data extraction form will be first piloted using a couple of the studies. Following the piloting, necessary amendments as required will be made to the extraction form and thereafter will be used for data extraction of all the studies. The data to be extracted will include: key characteristics of the included studies and study participants

For peer review only - http://bmjopen.bmj.com/site/about/guidelines.xhtml

(e.g. age, gender, family history of allergy/asthma, etc), study setting, study design, details of exposures, outcomes, statistical approach to the analysis of study data, missing data, and details of study findings. Author(s) of included studies will be contacted to request for additional data where such data are missing from the studies. Any discrepancies between reviewers in the data extraction will be resolved by either consensus or a third reviewer will arbitrate.

Risk of bias in individual studies

Risk of bias in individual studies will be undertaken by two independent reviewers using the Effective Public Health Practice Project (EPHPP) quality assessment tool. The EPHPP tool has six domains used to assess the potential risk of bias in each study, including selection bias, study design, confounders, blinding, data collection method, and withdrawals and dropouts. Each of these domains is rated as strong, moderate, or weak. Based on this domain rating, a global rating that integrates all the domain rating is determined for each study. Any discrepancies between reviewers in the risk of bias assessment will be resolved by either consensus or a third reviewer will arbitrate.

Data synthesis

Descriptive data from all studies will be presented in a table of characteristics. A narrative synthesis of the collected data across all studies will be undertaken. In addition, we will perform meta-analysis using random-effects models for studies judged to be sufficiently homogenous with regards to similarity in methods, study population, study design, exposure measures and assessment, and outcomes and assessment. Heterogeneity will be assessed using the I-squared statistic. We will undertake subgroup analysis to explore any potential differences in the estimates of the association between farm living and the study outcomes by age (children vs adults), world region (high income vs low and middle-income countries), and setting of the farm (rural vs urban). With sufficient amount of studies, we will perform meta-regression to further explore the underlying reasons for any potential heterogeneity in estimates between studies. Sensitivity analysis will be performed to explore any potential scenario that can change the conclusion of our findings, e.g. by excluding all low-quality studies from the meta-analysis and evaluate whether the results from high-quality studies differ from all studies included together. Publication bias will be explored using funnel plots and by calculating the Begg and Egger test[21-22]. The meta-analysis will be undertaken in RevMan (Review Manager 5.4). All this will be expressed with a 95 % confidence interval (CI).

For peer review only - http://bmjopen.bmj.com/site/about/guidelines.xhtml

Ethics and data management plan

As the study involves only the collection and analysis of data from already published literature, no ethics approval is required. All study data will be handled only by the researchers involved in the study and if relevant will be made open in the journal where the systematic review results will be published.

Patient and public involvement

No patient or members of the public was involved in the conception of this study and the development of the study protocol.

Dissemination

The study findings will be presented at scientific meetings related to the field of asthma and allergy and will be published in an international peer-reviewed scientific journal.

Consent for publication: Not applicable

Contributorship statement

BIN conceived the idea for this study. *JK* and *OO* wrote the first draft of the manuscript. All authors contributed to subsequent drafts, provided critical feedback, refinement of the study protocol and approved the final manuscript. BIN is the guarantor of the paper.

Competing interests : All the authors declare no competing interest

Funding: There are no funders to report for this submission (None)

Data sharing statement: Data sharing not applicable as no datasets generated and/or analysed for this study

Word count: 2114

For peer review only - http://bmjopen.bmj.com/site/about/guidelines.xhtml

REFERENCE

1. GBD 2015 Chronic Respiratory Disease Collaborators. Global, regional, and national deaths, prevalence, disability-adjusted life years, and years lived with disability for chronic obstructive pulmonary disease and asthma, 1990–2015: a systematic analysis for the Global Burden of Disease Study 2015. *The Lancet. Respiratory Medicine*. 2017 Sep;5(9):691.
2. Patelarou E, Tzanakis N, Kelly FJ. Exposure to indoor pollutants and wheeze and asthma development during early childhood. *International journal of environmental research and public health.* 2015 Apr;12(4):3993-4017.
3. Subbarao P, Becker A, Brook JR, Daley D, Mandhane PJ, Miller GE, Turvey SE, Sears MR. Epidemiology of asthma: risk factors for development. *Expert review of clinical immunology*. 2009 Jan 1;5(1):77-95.
4. World Health Organization (WHO). World Health Organization Asthma Fact sheet No. 307 [Internet]. Geneva: WHO; c2017 [cited 2018 Jan 17].
5. Blumenthal MN. Genetic, epigenetic, and environmental factors in asthma and allergy. *Annals of Allergy, Asthma & Immunology*. 2012 Feb 1;108(2):69-73.
6. Murrison LB, Brandt EB, Myers JB, Hershey GK. Environmental exposures and mechanisms in allergy and asthma development. *The Journal of clinical investigation*. 2019 Apr 1;129(4):1504-15.
7. Kirjavainen PV, Karvonen AM, Adams RI, Täubel M, Roponen M, Tuoresmäki P, Loss G, Jayaprakash B, Depner M, Ege MJ, Renz H. Farm-like indoor microbiota in non-farm homes protects children from asthma development. *Nature medicine.* 2019 Jul;25(7):1089-95.
8. Von Mutius E, Vercelli D. Farm living: effects on childhood asthma and allergy. *Nature Reviews Immunology.* 2010 Dec;10(12):861-8.
9. Stein MM, Hrusch CL, Gozdz J, Igartua C, Pivniouk V, Murray SE, Ledford JG, Marques dos Santos M, Anderson RL, Metwali N, Neilson JW. Innate immunity and asthma risk in Amish and Hutterite farm children. *New England journal of medicine.* 2016 Aug 4;375(5):411-21.
10. Ngajilo D, Adams S, Jeebhay MF. Occupational allergy and asthma in tobacco farmers: a review of literature. *Current Allergy & Clinical Immunology.* 2018 Jun 1;31(2):88-95.
11. Wlasiuk G, Vercelli D. The farm effect, or: when, what and how a farming environment protects from asthma and allergic disease. *Current opinion in allergy and clinical immunology.* 2012 Oct 1;12(5):461-6.
12. Brooks C, Pearce N, Douwes J. The hygiene hypothesis in allergy and asthma: an update. *Current opinion in allergy and clinical immunology.* 2013 Feb 1;13(1):70-7.
13. Heederik D, von Mutius E. Does diversity of environmental microbial exposure matter for the

For peer review only - http://bmjopen.bmj.com/site/about/guidelines.xhtml

occurrence of allergy and asthma?. *Journal of Allergy and Clinical Immunology.* 2012 Jul 1;130(1):44-50.

14. Ege MJ, Frei R, Bieli C, Schram-Bijkerk D, Waser M, Benz MR, Weiss G, Nyberg F, van Hage M, Pershagen G, Brunekreef B. Not all farming environments protect against the development of asthma and wheeze in children. Journal of Allergy and Clinical Immunology. 2007 May 1;119(5):1140-7.
15. Loss G, Apprich S, Waser M, Kneifel W, Genuneit J, Büchele G, Weber J, Sozanska B, Danielewicz H, Horak E, van Neerven RJ. The protective effect of farm milk consumption on childhood asthma and atopy: the GABRIELA study. *Journal of Allergy and Clinical Immunology.* 2011 Oct 1;128(4):766-73.
16. Frei R, Ferstl R, Roduit C, Ziegler M, Schiavi E, Barcik W, Rodriguez-Perez N, Wirz OF, Wawrzyniak M, Pugin B, Nehrbass D. Exposure to nonmicrobial N-glycolylneuraminic acid protects farmers' children against airway inflammation and colitis. *Journal of Allergy and Clinical Immunology.* 2018 Jan 1;141(1):382-90.
17. Scano G, Spinelli A, Duranti R, Gigliotti F, Turco R, Stendardi L, Emmi L, Arcangeli P. Effects of histamine and reproterol inhalation on mouth occlusion pressure in patients with bronchial asthma. *International Journal of Clinical Pharmacology, Therapy, and Toxicology.* 1986 Sep 1;24(9):490-5.
18. Thompson RH, Dicks D, Kramer SA. Clinical manifestations and functional outcomes in children with eosinophilic cystitis. *The Journal of urology.* 2005 Dec;174(6):2347-9.
19. Genuneit J. Exposure to farming environments in childhood and asthma and wheeze in rural populations: a systematic review with meta-analysis. *Pediatric Allergy and Immunology*. 2012 Sep;23(6):509-18.
20. Campbell JD, Brooks M, Hosokawa P, Robinson J, Song L, Krieger J. Community health worker home visits for Medicaid-enrolled children with asthma: effects on asthma outcomes and costs. *American Journal of Public Health*. 2015 Nov;105(11):2366-72.
21. Begg CB, Mazumdar M. Operating characteristics of a rank correlation test for publication bias. *Biometrics.* 1994 Dec 1:1088-101.
22. Egger M, Smith GD, Schneider M, Minder C. Bias in meta-analysis detected by a simple, graphical test. *BMJ.* 1997 Sep 13;315(7109):629-34.

For peer review only - http://bmjopen.bmj.com/site/about/guidelines.xhtml

APPENDIX 1

PUBMED SEARCH STRATEGY

CONCEPT 1: Farm (living)

"Farms"[Mesh] OR Farmland OR “farm living” OR farm* OR “crop farmer” OR “crop farmers” OR “crop farm” OR “crop farming” OR husbandar* OR animal* OR “animal rearing” OR farmstead* OR “ranch” OR “plantation” OR “croft” OR “shamba”.

CONCEPT 2: Asthma and allergy outcomes

Asthma[mh] OR Asthma, Exercise-Induced[mh] OR Respiratory Hypersensitivity[mh] OR bronchial asthma*[tiab] OR exercise-induced asthma*[tiab] OR asthma*[tiab] OR respiratory hypersensitivit*[tiab] OR airway hyper responsiveness[tiab] OR airway hyper-responsiveness[tiab] OR respiratory hyper responsiveness[tiab] OR respiratory hyper-responsiveness[tiab] OR wheez*[tiab] OR Hypersensitivity[mh] OR Hypersensitivity, Immediate[mh] OR Hypersensitivity, Delayed[mh] OR Allergy and Immunology[mh] OR immediate hypersensitivit*[tiab] OR delayed hypersensitivit*[tiab] OR hypersensitivit*[tiab] OR IgE-mediated hypersensitivit*[tiab] OR type I hypersensitivit*[tiab] OR type IV hypersensitivit*[tiab] OR atopic sensitization[tiab] OR atop*[tiab] OR allergic sensitization[tiab] OR allergic disease*[tiab] OR allerg*[tiab] OR Dermatitis, Atopic[mh] OR Eczema[mh] OR Angioedema[mh] OR Anaphylaxis[mh] OR Urticaria[mh] OR atopic dermatitis[tiab] OR dermatitis[tiab] OR atopic eczema[tiab] OR eczema[tiab] OR urticari*[tiab] OR anaphyla*[tiab] OR quincke edema[tiab angioedema[tiab] OR hives[tiab] OR Food Hypersensitivity[mh] OR food hypersensitivit*[tiab] OR food allerg*[tiab] OR egg allerg*[tiab] OR egg hypersensitivit*[tiab] OR milk allerg*[tiab] OR milk hypersensitivit*[tiab] OR shellfish allerg*[tiab] OR shellfish hypersensitivit*[tiab] OR wheat allerg*[tiab] OR wheat hypersensitivit*[tiab] OR nut allerg*[tiab] OR nut hypersensitivit*[tiab] OR peanut allerg*[tiab] OR peanut hypersensitivit*[tiab] OR Rhinitis, Allergic[mh] OR Rhinitis, Allergic, Seasonal[mh] OR Rhinitis, Allergic, Perennial[mh] OR Rhinitis[mh] OR Conjunctivitis, Allergic[mh] OR allergic rhinoconjunctiviti*[tiab] OR rhinoconjunctiviti*[tiab] OR allergic rhiniti*[tiab] OR rhiniti*[tiab] OR seasonal allergic rhiniti*[tiab] OR perennial allergic rhiniti*[tiab] OR allergic conjunctiviti*[tiab] OR hay fever[tiab] OR hayfever

For peer review only

Reporting checklist for protocol of a systematic review.

Based on the PRISMA-P guidelines.

Instructions to authors

Complete this checklist by entering the page numbers from your manuscript where readers will find each of the items listed below.

Your article may not currently address all the items on the checklist. Please modify your text to include the missing information. If you are certain that an item does not apply, please write "n/a" and provide a short explanation.

Upload your completed checklist as an extra file when you submit to a journal.

In your methods section, say that you used the PRISMA-Preporting guidelines, and cite them as:

Moher D, Shamseer L, Clarke M, Ghersi D, Liberati A, Petticrew M, Shekelle P, Stewart LA. Preferred Reporting Items for Systematic Review and Meta-Analysis Protocols (PRISMA-P) 2015 statement. Syst Rev. 2015;4(1):1.

For peer review only

		Reporting Item	Page Number
**Title**			
Identification	#1a	Identify the report as a protocol of a systematic review	1
Update	#1b	If the protocol is for an update of a previous systematic review, identify as such	N/A

**Registration**			
	#2	If registered, provide the name of the registry (such as PROSPERO) and registration number	2 & 6
**Authors**			
Contact	#3a	Provide name, institutional affiliation, e-mail address of all protocol authors; provide physical mailing address of corresponding author	1
Contribution	#3b	Describe contributions of protocol authors and identify the guarantor of the review	1
**Amendments**			
	#4	If the protocol represents an amendment of a previously completed or published protocol, identify as such and list changes; otherwise, state plan for documenting important protocol amendments	N/A
**Support**			
Sources	#5a	Indicate sources of financial or other support for the review	8
Sponsor	#5b	Provide name for the review funder and / or sponsor	8
Role of sponsor or funder	#5c	Describe roles of funder(s), sponsor(s), and / or institution(s), if any, in developing the protocol	8
**Introduction**			

For peer review only

Rationale	#6	Describe the rationale for the review in the context of what is already known	3 & 4
Objectives	#7	Provide an explicit statement of the question(s) the review will address with reference to participants, interventions, comparators, and outcomes (PICO)	4 & 5
**Methods**			
Eligibility criteria	#8	Specify the study characteristics (such as PICO, study design, setting, time frame) and report characteristics (such as years considered, language, publication status) to be used as criteria for eligibility for the review	5
Information sources	#9	Describe all intended information sources (such as electronic databases, contact with study authors, trial registers or other grey literature sources) with planned dates of coverage	6
Search strategy	#10	Present draft of search strategy to be used for at least one electronic database, including planned limits, such that it could be repeated	Apendix1
Study records - data management	#11a	Describe the mechanism(s) that will be used to manage records and data throughout the review	6 & 8
Study records - selection process	#11b	State the process that will be used for selecting studies (such as two independent reviewers) through each phase of the review (that is, screening, eligibility and inclusion in meta-analysis)	6

For peer review only

Study records - data collection process	#11c	Describe planned method of extracting data from reports (such as piloting forms, done independently, in duplicate), any processes for obtaining and confirming data from investigators	6 & 7
Data items	#12	List and define all variables for which data will be sought (such as PICO items, funding sources), any pre-planned data assumptions and simplifications	4 & 5
Outcomes and prioritization	#13	List and define all outcomes for which data will be sought, including prioritization of main and additional outcomes, with rationale	5
Risk of bias in individual studies	#14	Describe anticipated methods for assessing risk of bias of individual studies, including whether this will be done at the outcome or study level, or both; state how this information will be used in data synthesis	7
Data synthesis	#15a	Describe criteria under which study data will be quantitatively synthesised	7
Data synthesis	#15b	If data are appropriate for quantitative synthesis, describe planned summary measures, methods of handling data and methods of combining data from studies, including any planned exploration of consistency (such as I2, Kendall's τ)	7
Data synthesis	#15c	Describe any proposed additional analyses (such as sensitivity or subgroup analyses, meta-regression)	7

For peer review only

Data synthesis	#15d	If quantitative synthesis is not appropriate, describe the type of summary planned	n/a
Meta-bias(es)	#16	Specify any planned assessment of meta-bias(es) (such as publication bias across studies, selective reporting within studies)	7
Confidence in cumulative evidence	#17	Describe how the strength of the body of evidence will be assessed (such as GRADE)	7 & 8

The PRISMA-P checklist is distributed under the terms of the Creative Commons Attribution License CC-BY 4.0. This checklist was completed on 05. January 2021 using https://www.goodreports.org/, a tool made by the EQUATOR Network in collaboration with Penelope.ai

For peer review only

BMJ Open

Farm living and risk of asthma, atopic eczema, respiratory and food allergy: protocol for a systematic review and meta-analysis.

Journal:	*BMJ Open*
Manuscript ID	bmjopen-2021-048736.R1
Article Type:	Protocol
Date Submitted by the Author:	24-Sep-2021
Complete List of Authors:	Kuya, Johnstone; Africa Development and Strategy Centre, Public health<br>Omoronyia, Ogban; University of Calabar, Community Medicine<br>Fokukora, Theopista; Cavendish University, Department of Public Health<br>Emmanuel, Aduroja Posi; University of Ibadan, Department of Health Promotion and Education<br>Ewemooje, Olusegun; Federal University of Technology Akure, Department of Statistics<br>Soyege, Adewumni ; Babcock University, Department of Public Health<br>Ngo, Valery; University of Gothenburg, Department of Global Health<br>Anyiam, Felix Emeka; University of Port Harcourt<br>Ackah, Josephine Akua; University of Cape Coast, Department of Population and Health<br>Ossai-Chidi, Linus; University of Port Harcourt Teaching Hospital, Department of Medical Microbiology<br>Manuel, Beatriz; Eduardo Mondlane University, Department of Community Health<br>Okondu, Ogechukwu; Babcock University, Department of Public Health<br>Nwaru, Bright; University of Gothenburg
**Primary Subject Heading**:	Respiratory medicine
Secondary Subject Heading:	Epidemiology, Public health
Keywords:	Asthma < THORACIC MEDICINE, Allergy < THORACIC MEDICINE, RESPIRATORY MEDICINE (see Thoracic Medicine), PUBLIC HEALTH, EPIDEMIOLOGY

SCHOLARONE™
Manuscripts

Farm living and risk of asthma, atopic eczema, respiratory and food allergy: protocol for a systematic review and meta-analysis.

Johnstone Kuya[1], Ogban Omoronyia[2], Theopista Fokukora[3], Aduroja Posi Emmanuel [4] Olusegun Sunday Ewemooje[5] Adewumni O. Soyege [6], Valery Ngo[7], Felix Emeka Anyiam [8], Josephine Akua Ackah[9], Linus Ossai-Chidi[10], Beatriz Manuel[11], Okondu Ogechukwu Emmanuel [12], Bright I. Nwaru[13]

Author affiliations
[1]Africa Development and Strategy Centre, Nairobi, Kenya.
2 Department of Community Medicine, University of Calabar, Calabar, Nigeria
[3]Department of Public Health, Faculty of Science and Technology, Cavendish University, Kampala, Uganda
[4]Department of Health Promotion and Education, University of Ibadan, Ibadan, Nigeria.
[5]Department of Statistics, Federal University of Technology, Akure, Nigeria.
[6]Department of Public Health, School of Public and Allied Health, Babcock University, Ilishan-Remo, Nigeria.
[7]Department of Global Health, School of Public Health and Community Medicine, The Sahlgrenska Academy, University of Gothenburg, Gothenburg, Sweden.
[8]Centre for Health and Development (CHD), University of Port Harcourt, Port Harcourt, Nigeria
[9] Department of Population and Health, Faculty of Social Sciences, University of Cape Coast, Ghana
[10] Department of Medical Microbiology, University of Port Harcourt Teaching Hospital, Rivers State. Nigeria
[11] Department of Community Health, Faculty of Medicine, Eduardo Mondlane University, Maputo Mozambique.
[12] Department of Public Health, School of Public and Allied Health, Babcock University, Ilishan-Remo, Nigeria
[13]Krefting Research Centre, Institute of Medicine, Sahlgrenska Academy, University of Gothenburg, Gothenburg, Sweden

Correspondence:

Bright Nwaru
Krefting Research Centre
Institute of Medicine
University of Gothenburg
+46 31 786 6718
bright.nwaru@gu.se

For peer review only

ABSTRACT

Introduction: Asthma and allergic disorders are of global concern and risk for their development results from the interplay of genetic and environmental factors. Among numerous environmental factors identified to influence the disease risk, the role of exposure to a farming environment has gained interest in recent times, but the underlying evidence is conflicting. The review aim to comprehensively identify, critically appraise, and synthesize evidence from studies investigating the association between farm living and risk of asthma and allergic diseases in children and adults.

Methods and Analysis: We will identify relevant analytical observational epidemiological studies, including cross-sectional, case-control and cohort studies, by searching PubMed, Cochrane Library, Google Scholar, CINAHL, WHO Global Health Library, Web of Science, Scopus, and EMBASE. Screening of identified records, data extraction from eligible studies and risk of bias assessment of eligible studies will be independently undertaken by two reviewers, with arbitration by a third reviewer. The Effective Public Health Practice Project will be employed for the risk of bias assessment. Estimates from studies judged to be clinically, methodologically, and statistically homogeneous will be synthesised using random-effects meta-analysis. Heterogeneity will be assessed using the I-squared statistic. We will consider objectively measured or self-reported asthma, atopic dermatitis/eczema, allergic rhinitis, wheeze, and food allergy as primary outcomes.

Ethics and dissemination: As this study is based solely on the published literature, no ethics approval is required. The study findings will be presented at scientific meetings related to the field of asthma and allergy and will be published in an international peer-reviewed scientific journal.

PROSPERO registration number: CRD42020208805

Strengths and Limitations of this Study:

- Findings from this systematic review will provide a comprehensive and most contemporaneous synthesis of the underlying evidence linking exposure to a farming environment to risk of asthma and allergic diseases since the latest publication of the previous reviews was five years ago.
- This systematic review protocol follows the Preferred Reporting Items for Systematic Review and Meta-Analysis Protocols guidelines.
- The identification of studies from leading medical and public health databases, with no geographical or language limitations, will advance the import of this evidence synthesis across settings.
- We anticipate that the definition of farm exposure and asthma and allergic outcomes will vary between studies, thus posing a challenge in deriving pooled estimates of effect.

KEYWORDS: Systematic review; meta-analysis, Asthma, wheeze, atopic dermatitis, atopic sensitization, farm living, farming, allergic rhinitis, food allergy.

For peer review only - http://bmjopen.bmj.com/site/about/guidelines.xhtml

BACKGROUND

Globally, there is high morbidity due to asthma and allergic diseases[1]. This group of immunologic conditions have continued to contribute significantly to the burden of disease and years lost due to disability in diverse global settings[1]. Currently, an estimated 339 million people have asthma, which represents the most common chronic disease among children[2]. The development of asthma and allergic diseases results from the interplay of genetic and environmental factors[3].

Numerous environmental factors linked to the risk of asthma and allergic diseases have been identified, among which include household exposures (e.g., dust mites, household dampness, indoor air pollution, moulds, and domestic pets) and non-household exposures (e.g., respiratory infections, tobacco smoke, outdoor air pollution, and exposure to a farming environment)[4]. However, findings from available studies on the influence of these environmental factors are conflicting[5-7]. With a specific focus on the role of the farming environment, over the last three decades, several studies have reported protection for atopy, allergic disease, and asthma[8], while some studies have also reported that exposure to the farming environment may increase the risk of the onset of the diseases[9,10]. While most of these studies have focused on exposure to a farming environment during early childhood and asthma and allergy in childhood, there are also some studies in adults.

The amount and variety of microbial exposure from farm living are thought to be a key mechanism through which the farming environment influences the risk of asthma and allergies[11]. Greater microbial exposure, characteristic of the more unhygienic farm environment, stimulates the development of a more effective immune system[12]. Microbial exposure helps the immune system to learn to efficiently avoid over-reaction to potentially harmless substances that may have been perceived as allergens[11]. However, the effect of microbial exposure may differ according to farm living environment or farming activities[13]. A large-scale multicentre cross-sectional study among rural school children in five European countries found that pig farming, haying, consumption of farm milk, and dwelling in the farm were associated with substantially reduced risk of asthma compared to wheeze[14]. The high proportion of whey protein found in farm milk is thought to initiate the process of more effective regulation of the immune system to prevent over-reactivity to diverse substances[15]. Furthermore, sialic acid, more commonly found in farm animals and their products, is thought to contribute to antibody-mediated regulation of inflammatory reactions to substances[16]. Several systematic reviews have been published that summarized the available evidence linking the farming environment to the risk of asthma and allergy. Dicks and colleagues[17, 18] in their review

For peer review only

revealed that habitation of children in an area with intensive farming was associated with an increased risk of being asthmatic. The review considered asthma and wheeze as the only outcomes, but importantly, it was based on searches made only in PubMed and was undertaken by only one person. Without consideration of other allergic outcomes other than asthma and wheeze does not give a comprehensive overview of the underlying evidence. Searching only one database of several databases that collect scientific research also indicates that the generated evidence is limited, given that the overlap between PubMed and other research databases is only partial. A systematic review should provide the highest evidence on a research topic and therefore the process needs to be transparent and reproducible, with at least two reviewers going through the processes of the review being a mandatory requirement; however, Genuneit[19] failed in this aspect as it was undertaken only by one person. The systematic review by Campbell and colleagues[20] focused only on objective measures of atopy (based on skin prick test [SPT] and immunoglobulin E [IgE] measurements) and was also based on studies retrieved only from PubMed. Similar limitations affected this review, in that it investigated a small sub-set of atopic outcomes and performed a limited search of available literature. Including both objective measures and subjective measures in the assessment of allergy, which are heterogeneous in nature, will provide a clearer insight into the impact of farm living on these outcomes.

To gain a clearer and comprehensive appreciation of the underlying evidence linking exposure to a farming environment to risk of asthma and allergic diseases, the noted limitations in previous systematic reviews need to be overcome: there should be an exhaustive set of outcomes included; there should be an exhaustive search of the literature; and the review processes should be transparent and reproducible, with at least two people doing the review. Moreover, since the publication of the previous reviews, the latest published five years ago, there have been several studies now published on the topic. These studies need to be integrated into the evidence synthesis to provide a contemporaneous synthesis of the evidence base.

AIMS AND OBJECTIVES

By overcoming limitations of previous systematic reviews, including limited outcomes studied, limited databases search, sole reviewer, and a long time since their conduct, we aim to comprehensively identify, critically appraise, and synthesize evidence from studies investigating the association between farm living and risk of asthma and allergic diseases in children and adults.

For peer review only

Specific objectives are to synthesize the evidence on:

1. The association between farm living and the risk of the onset of asthma and allergic diseases in children and adults.
2. The association between farm living and the risk of outcomes (e.g., indicators of exacerbation and severity) of asthma and allergic diseases in children and adults with already established disease.
3. Whether the association between farm living and risk of asthma and allergy depends on the type of farming environment (e.g., pig farmers vs cattle rearers, small vs large-scale farmers).
4. Whether the association between farm living and risk of asthma and allergy differs between geographical regions of the world (e.g., high-income vs low and middle-income countries).

METHODS

Study eligibility criteria

Type of studies: We will include analytical observational epidemiological studies (cross-sectional, case-control and cohort studies). We will exclude clinical case studies, case-series discussion papers, non-research letters and editorials, and animal studies.

Types of participants

We will include studies that included participants of any age, in which the association between farm living and asthma and allergic diseases have been investigated, regardless of gender, age and nationality. Children will be defined as those below the age of 18 years and adults as those 18 years and above.

Types of exposure

Studies that have investigated the role of farm living, regardless of the type of farm environment and the place of farming (rural versus urban). We will not consider the specific constituents of the farm (e.g., bioaerosols) and we will define farm living as having a residence in a farming environment, either as an active farmer or passive farmer (i.e., living on a farm but not a farmer by yourself).

Types of outcome measures

As primary outcomes, we will consider objectively-measured or self-reported asthma, atopic dermatitis/eczema, allergic rhinitis, wheeze, and food allergy. Our secondary outcomes will include atopic sensitization measured by skin prick test or antigen-specific immunoglobulin E; indicators of

For peer review only

disease severity and measures of quality of life; asthma exacerbations; use of medications for respective allergic diseases and asthma; hospitalisation for asthma; indicators of airway function including peak expiratory flow, forced expiratory volume in 1 second, forced vital capacity, forced expiratory flow rate, or other pulmonary function tests [oscillometry or exhaled nitric oxide analysis]).

Information sources

Database searches

Relevant studies/articles from the onset of the following electronic databases till date will be sought from: PubMed, Cochrane Library, Google Scholar, CINAHL, WHO Global Health Library, Web of Science, Scopus, and EMBASE. There will be no restriction on language, and we will endeavour to translate papers not published in English. We will supplement the records retrieved from the respective databases by contacting previous authors on the topic to identify any studies our searches might have missed.

Search strategy

Search strategies covering concepts and synonyms of farm living and the study outcomes (refer to the appendix of search strategies) will be adapted to search the other databases.

Screening of retrieved literature

The literature retrieved from the databases will be transferred to Endnote for removal of duplicate papers; thereafter the papers will be exported to Rayan for further screening. Two reviewers will separately evaluate the titles and abstracts of the de-duplicated articles based on the study inclusion and exclusion criteria. The full texts of eligible articles will be collected and further critically reviewed by the two reviewers for inclusion. Any differences between the two reviewers in the evaluation processes will be settled by consensus, and a third reviewer will arbitrate any disagreement. The Preferred Reporting Items for Systematic Reviews and Meta-Analysis PRISMA flow chart will be used to report the screening process.(21) The MOOSE guideline will be used in reporting the results from the meta-analyses.(22)

Registration and reporting

This study protocol has been registered with the University of York Centre for Reviews and Dissemination International prospective register of systematic reviews (PROSPERO number

For peer review only - http://bmjopen.bmj.com/site/about/guidelines.xhtml

CRD42020208805). The protocol is reported according to the PRISMA-P guidelines for reporting of systematic review protocols, awaiting registration number.

Data extraction

A standardized data extraction form will be developed and will be used by at least two members of the review team to independently extract relevant study data from the full-text papers included in the systematic review. Before full use with all included studies, the data extraction form will be first piloted using a couple of the studies. Following the piloting, necessary amendments as required will be made to the extraction form and thereafter will be used for data extraction of all the studies. The data to be extracted will include key characteristics of the included studies and study participants (e.g., age, gender, family history of allergy/asthma, etc), study setting, study design, details of exposures, outcomes, statistical approach to the analysis of study data, missing data, and details of study findings. Author(s) of included studies will be contacted to request additional data where such data are missing from the studies. Any discrepancies between reviewers in the data extraction will be resolved by either consensus or a third reviewer will arbitrate.

Risk of bias in individual studies

The risk of bias in individual studies will be undertaken by two independent reviewers using the Effective Public Health Practice Project (EPHPP) quality assessment tool. The EPHPP tool has six domains used to assess the potential risk of bias in each study, including selection bias, study design, confounders, blinding, data collection method, and withdrawals and dropouts. Each of these domains is rated as strong, moderate, or weak. Based on this domain rating, a global rating that integrates all the domain ratings is determined for each study. Any discrepancies between reviewers in the risk of bias assessment will be resolved by either consensus or a third reviewer will arbitrate.

Data synthesis

Descriptive data from all studies will be presented in a table of characteristics. A narrative synthesis of the collected data across all studies will be undertaken. In addition, we will perform meta-analysis using random-effects models for studies judged to be sufficiently homogenous with regards to a similarity in methods, study population, study design, exposure measures and assessment, and outcomes and assessment. Heterogeneity will be assessed using the I-squared statistic. We will undertake subgroup analysis to explore any potential differences in the estimates of the association between farm living and the study outcomes by age (children vs adults), world region (high income vs low and middle-income countries) and setting of the farm (rural vs urban). With a sufficient amount

For peer review only

For peer review only - http://bmjopen.bmj.com/site/about/guidelines.xhtml

of studies, we will perform meta-regression to further explore the underlying reasons for any potential heterogeneity in estimates between studies. Sensitivity analysis will be performed to explore any potential scenario that can change the conclusion of our findings, e.g., by excluding all low-quality studies from the meta-analysis and evaluate whether the results from high-quality studies differ from all studies included together. Publication bias will be explored using funnel plots and by calculating the Begg and Egger test[23-24]. The meta-analysis will be undertaken in RevMan (Review Manager 5.4). All this will be expressed with a 95 % confidence interval (CI).

Ethics and data management plan

As the study involves only the collection and analysis of data from already published literature, no ethics approval is required. All study data will be handled only by the researchers involved in the study and if relevant will be made open in the journal where the systematic review results will be published.

Patient and public involvement

No patient or members of the public was involved in the conception of this study and the development of the study protocol.

Dissemination

The study findings will be presented at scientific meetings related to the field of asthma and allergy and will be published in an international peer-reviewed scientific journal.

Consent for publication: Not applicable

Contributorship statement

<u>**Authors'**</u>

Johnstone Kuya[1], Ogban Omoronyia[2], Theopista Fokukora[3], Aduroja Posi Emmanuel [4] Olusegun Sunday Ewemooje[5] Adewumni O. Soyege [6], Valery Ngo[7], Felix Emeka Anyiam [8], Josephine Akua Ackah[9], Linus Ossai-Chidi[10], Beatriz Manuel[11], Okondu Ogechukwu Emmanuel [12], Bright I. Nwaru[13]

BIN conceived of the presented idea of the study and supervised the project. *JK* and *OO* wrote the first draft of the manuscript with TF, APE, OSE, and AOS. *OSE, APE, FEA*, *OOE* with assistance from VN, *BM, LO-C and JA* provided methodological and statistical expertise. All authors contributed to subsequent drafts, provided critical feedback, refinement of the study protocol and approved the final manuscript." BIN is the guarantor of the paper.

For peer review only - http://bmjopen.bmj.com/site/about/guidelines.xhtml

Competing interests: All the authors declare no competing interest

Funding: There are no funders to report for this submission (None)

Data sharing statement: Data sharing is not applicable as no datasets generated and/or analysed for this study

Word count: 2644

REFERENCE

1. GBD 2015 Chronic Respiratory Disease Collaborators. Global, regional, and national deaths, prevalence, disability-adjusted life years, and years lived with disability for chronic obstructive pulmonary disease and asthma, 1990–2015: a systematic analysis for the Global Burden of Disease Study 2015. *The Lancet. Respiratory Medicine*. 2017 Sep;5(9):691.
2. Patelarou E, Tzanakis N, Kelly FJ. Exposure to indoor pollutants and wheeze and asthma development during early childhood. *International journal of environmental research and public health.* 2015 Apr;12(4):3993-4017.
3. Subbarao P, Becker A, Brook JR, Daley D, Mandhane PJ, Miller GE, Turvey SE, Sears MR. Epidemiology of asthma: risk factors for development. *Expert review of clinical immunology*. 2009 Jan 1;5(1):77-95.
4. World Health Organization (WHO). World Health Organization Asthma Fact sheet No. 307 [Internet]. Geneva: WHO; c2017 [cited 2018 Jan 17].
5. Blumenthal MN. Genetic, epigenetic, and environmental factors in asthma and allergy. *Annals of Allergy, Asthma & Immunology*. 2012 Feb 1;108(2):69-73.
6. Murrison LB, Brandt EB, Myers JB, Hershey GK. Environmental exposures and mechanisms in allergy and asthma development. *The Journal of clinical investigation*. 2019 Apr 1;129(4):1504-15.
7. Kirjavainen PV, Karvonen AM, Adams RI, Täubel M, Roponen M, Tuoresmäki P, Loss G, Jayaprakash B, Depner M, Ege MJ, Renz H. Farm-like indoor microbiota in non-farm homes protects children from asthma development. *Nature medicine.* 2019 Jul;25(7):1089-95.
8. Von Mutius E, Vercelli D. Farm living: effects on childhood asthma and allergy. *Nature Reviews Immunology.* 2010 Dec;10(12):861-8.

For peer review only

9. Stein MM, Hrusch CL, Gozdz J, Igartua C, Pivniouk V, Murray SE, Ledford JG, Marques dos Santos M, Anderson RL, Metwali N, Neilson JW. Innate immunity and asthma risk in Amish and Hutterite farm children. *New England journal of medicine.* 2016 Aug 4;375(5):411-21.
10. Ngajilo D, Adams S, Jeebhay MF. Occupational allergy and asthma in tobacco farmers: a review of literature. *Current Allergy & Clinical Immunology.* 2018 Jun 1;31(2):88-95.
11. Wlasiuk G, Vercelli D. The farm effect, or: when, what and how a farming environment protects from asthma and allergic disease. *Current opinion in allergy and clinical immunology.* 2012 Oct 1;12(5):461-6.
12. Brooks C, Pearce N, Douwes J. The hygiene hypothesis in allergy and asthma: an update. *Current opinion in allergy and clinical immunology.* 2013 Feb 1;13(1):70-7.
13. Heederik D, von Mutius E. Does diversity of environmental microbial exposure matter for the occurrence of allergy and asthma? *Journal of Allergy and Clinical Immunology.* 2012 Jul 1;130(1):44-50.
14. Ege MJ, Frei R, Bieli C, Schram-Bijkerk D, Waser M, Benz MR, Weiss G, Nyberg F, van Hage M, Pershagen G, Brunekreef B. Not all farming environments protect against the development of asthma and wheeze in children. Journal of Allergy and Clinical Immunology. 2007 May 1;119(5):1140-7.
15. Loss G, Apprich S, Waser M, Kneifel W, Genuneit J, Büchele G, Weber J, Sozanska B, Danielewicz H, Horak E, van Neerven RJ. The protective effect of farm milk consumption on childhood asthma and atopy: the GABRIELA study. *Journal of Allergy and Clinical Immunology.* 2011 Oct 1;128(4):766-73.
16. Frei R, Ferstl R, Roduit C, Ziegler M, Schiavi E, Barcik W, Rodriguez-Perez N, Wirz OF, Wawrzyniak M, Pugin B, Nehrbass D. Exposure to nonmicrobial N-glycolylneuraminic acid protects farmers' children against airway inflammation and colitis. *Journal of Allergy and Clinical Immunology.* 2018 Jan 1;141(1):382-90.
17. Scano G, Spinelli A, Duranti R, Gigliotti F, Turco R, Stendardi L, Emmi L, Arcangeli P. Effects of histamine and reproterol inhalation on mouth occlusion pressure in patients with bronchial asthma. *International Journal of Clinical Pharmacology, Therapy, and Toxicology.* 1986 Sep 1;24(9):490-5.
18. Thompson RH, Dicks D, Kramer SA. Clinical manifestations and functional outcomes in children with eosinophilic cystitis. *The Journal of urology.* 2005 Dec;174(6):2347-9.
19. Genuneit J. Exposure to farming environments in childhood and asthma and wheeze in rural populations: a systematic review with meta-analysis. *Pediatric Allergy and Immunology.* 2012 Sep;23(6):509-18.

For peer review only - http://bmjopen.bmj.com/site/about/guidelines.xhtml

20. Campbell JD, Brooks M, Hosokawa P, Robinson J, Song L, Krieger J. Community health worker home visits for Medicaid-enrolled children with asthma: effects on asthma outcomes and costs. *American Journal of Public Health*. 2015 Nov;105(11):2366-72.

21. Page MJ, McKenzie JE, Bossuyt PM, Boutron I, Hoffmann TC, Mulrow CD, Shamseer L, Tetzlaff JM, Akl EA, Brennan SE, Chou R, Glanville J, Grimshaw JM, Hróbjartsson A, Lalu MM, Li T, Loder EW, Mayo-Wilson E, McDonald S, McGuinness LA, Stewart LA, Thomas J, Tricco AC, Welch VA, Whiting P, Moher D. The PRISMA 2020 statement: an updated guideline for reporting systematic reviews. BMJ. 2021 Mar 29;372:n71. doi: 10.1136/bmj.n71. PMID: 33782057; PMCID: PMC8005924.

22. Stroup DF, Berlin JA, Morton SC, Olkin I, Williamson GD, Rennie D, Moher D, Becker BJ, Sipe TA, Thacker SB. Meta-analysis of observational studies in epidemiology: a proposal for reporting. Meta-analysis Of Observational Studies in Epidemiology (MOOSE) group. JAMA. 2000 Apr 19;283(15):2008-12. doi: 10.1001/jama.283.15.2008. PMID: 10789670.

23. Begg CB, Mazumdar M. Operating characteristics of a rank correlation test for publication bias. *Biometrics.* 1994 Dec 1:1088-101.

24. Egger M, Smith GD, Schneider M, Minder C. Bias in meta-analysis detected by a simple, graphical test. *BMJ.* 1997 Sep 13;315(7109):629-34.

For peer review only

For peer review only - http://bmjopen.bmj.com/site/about/guidelines.xhtml

APPENDIX 1

PUBMED SEARCH STRATEGY

CONCEPT 1: Farm (living)

"Farms"[Mesh] OR Farmland OR "farm living" OR farm* OR "crop farmer" OR "crop farmers" OR "crop farm" OR "crop farming" OR husbandar* OR animal* OR "animal rearing" OR farmstead* OR "ranch" OR "plantation" OR "croft" OR "shamba".

CONCEPT 2: Asthma and allergy outcomes

Asthma[mh] OR Asthma, Exercise-Induced[mh] OR Respiratory Hypersensitivity[mh] OR bronchial asthma*[tiab] OR exercise-induced asthma*[tiab] OR asthma*[tiab] OR respiratory hypersensitivit*[tiab] OR airway hyper responsiveness[tiab] OR airway hyper-responsiveness[tiab] OR respiratory hyper responsiveness[tiab] OR respiratory hyper-responsiveness[tiab] OR wheez*[tiab] OR Hypersensitivity[mh] OR Hypersensitivity, Immediate[mh] OR Hypersensitivity, Delayed[mh] OR Allergy and Immunology[mh] OR immediate hypersensitivit*[tiab] OR delayed hypersensitivit*[tiab] OR hypersensitivit*[tiab] OR IgE-mediated hypersensitivit*[tiab] OR type I hypersensitivit*[tiab] OR type IV hypersensitivit*[tiab] OR atopic sensitization[tiab] OR atop*[tiab] OR allergic sensitization[tiab] OR allergic disease*[tiab] OR allerg*[tiab] OR Dermatitis, Atopic[mh] OR Eczema[mh] OR Angioedema[mh] OR Anaphylaxis[mh] OR Urticaria[mh] OR atopic dermatitis[tiab] OR dermatitis[tiab] OR atopic eczema[tiab] OR eczema[tiab] OR urticari*[tiab] OR anaphyla*[tiab] OR quincke edema[tiab angioedema[tiab] OR hives[tiab] OR Food Hypersensitivity[mh] OR food hypersensitivit*[tiab] OR food allerg*[tiab] OR egg allerg*[tiab] OR egg hypersensitivit*[tiab] OR milk allerg*[tiab] OR milk hypersensitivit*[tiab] OR shellfish allerg*[tiab] OR shellfish hypersensitivit*[tiab] OR wheat allerg*[tiab] OR wheat hypersensitivit*[tiab] OR nut allerg*[tiab] OR nut hypersensitivit*[tiab] OR peanut allerg*[tiab] OR peanut hypersensitivit*[tiab] OR Rhinitis, Allergic[mh] OR Rhinitis, Allergic, Seasonal[mh] OR Rhinitis, Allergic, Perennial[mh] OR Rhinitis[mh] OR Conjunctivitis, Allergic[mh] OR allergic rhinoconjunctiviti*[tiab] OR rhinoconjunctiviti*[tiab] OR allergic rhiniti*[tiab] OR rhiniti*[tiab] OR seasonal allergic rhiniti*[tiab] OR perennial allergic rhiniti*[tiab] OR allergic conjunctiviti*[tiab] OR hay fever[tiab] OR hayfever

For peer review only

MOOSE (Meta-analyses Of Observational Studies in Epidemiology) Checklist

A reporting checklist for Authors, Editors, and Reviewers of Meta-analyses of Observational Studies. You must report the page number in your manuscript where you consider each of the items listed in this checklist. If you have not included this information, either revise your manuscript accordingly before submitting or note N/A.

Reporting Criteria	Reported (Yes/No)	Reported on Page No.
**Reporting of Background**		
Problem definition	Yes	3
Hypothesis statement	No	N/A
Description of Study Outcome(s)	Yes	5
Type of exposure or intervention used	Yes	5
Type of study design used	Yes	5
Study population	Yes	5
**Reporting of Search Strategy**		
Qualifications of searchers (eg, librarians and investigators)	No	N/A
Search strategy, including time period included in the synthesis and keywords	Yes	11
Effort to include all available studies, including contact with authors	Yes	7
Databases and registries searched	Yes	6
Search software used, name and version, including special features used (eg, explosion)	Yes	6
Use of hand searching (eg, reference lists of obtained articles)	No	N/A
List of citations located and those excluded, including justification	No	N/A
Method for addressing articles published in languages other than English	Yes	6
Method of handling abstracts and unpublished studies	No	N/A
Description of any contact with authors	No	N/A
**Reporting of Methods**		
Description of relevance or appropriateness of studies assembled for assessing the hypothesis to be tested	Yes	6
Rationale for the selection and coding of data (eg, sound clinical principles or convenience)	Yes	6,7,8
Documentation of how data were classified and coded (eg, multiple raters, blinding, and interrater reliability)	Yes	6,7,8
Assessment of confounding (eg, comparability of cases and controls in studies where appropriate	Yes	7

For peer review only

Reporting Criteria	Reported (Yes/No)	Reported on Page No.
Assessment of study quality, including blinding of quality assessors; stratification or regression on possible predictors of study results	Yes	7
Assessment of heterogeneity	Yes	7
Description of statistical methods (eg, complete description of fixed or random effects models, justification of whether the chosen models account for predictors of study results, dose-response models, or cumulative meta-analysis) in sufficient detail to be replicated	Yes	7
Provision of appropriate tables and graphics	No	N/A
**Reporting of Results**		
Table giving descriptive information for each study included	No	N/A
Results of sensitivity testing (eg, subgroup analysis)	Yes	7
Indication of statistical uncertainty of findings	No	N/A
**Reporting of Discussion**		
Quantitative assessment of bias (eg, publication bias)	Yes	7
Justification for exclusion (eg, exclusion of non–English-language citations)	Yes	5
Assessment of quality of included studies	Yes	7
**Reporting of Conclusions**		
Consideration of alternative explanations for observed results	Yes	8
Generalization of the conclusions (ie, appropriate for the data presented and within the domain of the literature review)	Yes	8
Guidelines for future research	No	N/A
Disclosure of funding source	Yes	8

Once you have completed this checklist, please save a copy and upload it as part of your submission. DO NOT include this checklist as part of the main manuscript document. It must be uploaded as a separate file.

For peer review only

Reporting checklist for protocol of a systematic review.

Based on the PRISMA-P guidelines.

Instructions to authors

Complete this checklist by entering the page numbers from your manuscript where readers will find each of the items listed below.

Your article may not currently address all the items on the checklist. Please modify your text to include the missing information. If you are certain that an item does not apply, please write "n/a" and provide a short explanation.

Upload your completed checklist as an extra file when you submit to a journal.

In your methods section, say that you used the PRISMA-Preporting guidelines, and cite them as:

Moher D, Shamseer L, Clarke M, Ghersi D, Liberati A, Petticrew M, Shekelle P, Stewart LA. Preferred Reporting Items for Systematic Review and Meta-Analysis Protocols (PRISMA-P) 2015 statement. Syst Rev. 2015;4(1):1.

		Reporting Item	Page Number
**Title**			
Identification	#1a	Identify the report as a protocol of a systematic review	1
Update	#1b	If the protocol is for an update of a previous systematic review, identify as such	N/A

For peer review only

**Registration**			
	#2	If registered, provide the name of the registry (such as PROSPERO) and registration number	2 & 6
**Authors**			
Contact	#3a	Provide name, institutional affiliation, e-mail address of all protocol authors; provide physical mailing address of corresponding author	1
Contribution	#3b	Describe contributions of protocol authors and identify the guarantor of the review	1
**Amendments**			
	#4	If the protocol represents an amendment of a previously completed or published protocol, identify as such and list changes; otherwise, state plan for documenting important protocol amendments	N/A
**Support**			
Sources	#5a	Indicate sources of financial or other support for the review	8
Sponsor	#5b	Provide name for the review funder and / or sponsor	8
Role of sponsor or funder	#5c	Describe roles of funder(s), sponsor(s), and / or institution(s), if any, in developing the protocol	8
**Introduction**			

For peer review only

Rationale	#6	Describe the rationale for the review in the context of what is already known	3 & 4
Objectives	#7	Provide an explicit statement of the question(s) the review will address with reference to participants, interventions, comparators, and outcomes (PICO)	4 & 5
**Methods**			
Eligibility criteria	#8	Specify the study characteristics (such as PICO, study design, setting, time frame) and report characteristics (such as years considered, language, publication status) to be used as criteria for eligibility for the review	5
Information sources	#9	Describe all intended information sources (such as electronic databases, contact with study authors, trial registers or other grey literature sources) with planned dates of coverage	6
Search strategy	#10	Present draft of search strategy to be used for at least one electronic database, including planned limits, such that it could be repeated	Apendix1
Study records - data management	#11a	Describe the mechanism(s) that will be used to manage records and data throughout the review	6 & 8
Study records - selection process	#11b	State the process that will be used for selecting studies (such as two independent reviewers) through each phase of the review (that is, screening, eligibility and inclusion in meta-analysis)	6

For peer review only

Study records - data collection process	#11c	Describe planned method of extracting data from reports (such as piloting forms, done independently, in duplicate), any processes for obtaining and confirming data from investigators	6 & 7
Data items	#12	List and define all variables for which data will be sought (such as PICO items, funding sources), any pre-planned data assumptions and simplifications	4 & 5
Outcomes and prioritization	#13	List and define all outcomes for which data will be sought, including prioritization of main and additional outcomes, with rationale	5
Risk of bias in individual studies	#14	Describe anticipated methods for assessing risk of bias of individual studies, including whether this will be done at the outcome or study level, or both; state how this information will be used in data synthesis	7
Data synthesis	#15a	Describe criteria under which study data will be quantitatively synthesised	7
Data synthesis	#15b	If data are appropriate for quantitative synthesis, describe planned summary measures, methods of handling data and methods of combining data from studies, including any planned exploration of consistency (such as I2, Kendall's τ)	7
Data synthesis	#15c	Describe any proposed additional analyses (such as sensitivity or subgroup analyses, meta-regression)	7

For peer review only

Data synthesis	#15d	If quantitative synthesis is not appropriate, describe the type of summary planned	n/a
Meta-bias(es)	#16	Specify any planned assessment of meta-bias(es) (such as publication bias across studies, selective reporting within studies)	7
Confidence in cumulative evidence	#17	Describe how the strength of the body of evidence will be assessed (such as GRADE)	7 & 8

The PRISMA-P checklist is distributed under the terms of the Creative Commons Attribution License CC-BY 4.0. This checklist was completed on 05. January 2021 using https://www.goodreports.org/, a tool made by the EQUATOR Network in collaboration with Penelope.ai

For peer review only
